# Characteristics of the aberrant pyramidal tract in comparison with the pyramidal tract in the human brain

**DOI:** 10.1186/1471-2202-12-108

**Published:** 2011-11-01

**Authors:** Hyeok Gyu Kwon, Su Min Son, Min Cheol Chang, Saeyoon Kim, Yong Hyun Kwon, Sung Ho Jang

**Affiliations:** 1 Department of Physical Medicine and Rehabilitation, College of Medicine, Yeungnam University, Daegu, Republic of Korea; 2 Department of Pediatrics, College of Medicine, Yeungnam University, Daegu, Republic of Korea; 3 Department of Physical Therapy, Yeungnam College of Science & Technology, Daegu, Republic of Korea

## Abstract

**Background:**

The aberrant pyramidal tract (APT) refers to the collateral pathway of the pyramidal tract (PT) through the medial lemniscus in the midbrain and pons. Using diffusion tensor tractography (DTT), we investigated the characteristics of the APT in comparison with the PT in the normal human brain.

**Results:**

In thirty-four (18.3%, right hemisphere: 20, left hemisphere: 14) of the 186 hemispheres, the APTs separated from the PT at the upper midbrain level, descended through the medial lemniscus from the midbrain to the pons, and then rejoined with the PT at the upper medulla. Nine (26.5%) of the 34 APTs were found to originate from the primary somatosensory cortex without a primary motor cortex origin. Values of fractional anisotropy (FA) and tract volume of the APT were lower than those of the PT (*P *< 0.05); however, no difference in mean diffusivity (MD) value was observed (*P >*0.05).

**Conclusion:**

We found that the APT has different characteristics, including less directionality, fewer neural fibers, and less origin from the primary motor cortex than the PT.

## Background

The pyramidal tract (PT) is a major neuronal pathway for mediation of voluntary movements in the human brain and has been known to have collateral pathways [1,2]. The aberrant pyramidal tract (APT) indicates the collateral pathway of the PT, which separates from the original PT at the level of the midbrain and the pons, and descends through the medial lemniscus [3-6]. Existence of the APT has been demonstrated by various methods, including pathological, electrophysiological, and radiological studies [3,6-10]. In addition, recently, the APT has been suggested as a motor recovery mechanism in stroke [11-14]. However, detailed characteristics of the APT have not been clearly elucidated.

Diffusion tensor tractography (DTT), which is derived from diffusion tensor imaging (DTI), allows visualization and estimation of the PT and APT in three dimensions [3,11,12]. Several DTT studies have reported on the presence of the APT in normal subjects or in patients with brain injury [3,11-14]. However, so far, little is known about the characteristics of the APT in the human brain.

In the current study, we investigated the characteristics of the APT in comparison with the PT in the normal human brain, using DTT.

## Results

In the brains of all subjects, PTs were found to originate from the primary sensori-motor cortex (SM1, anterior boundary: precentral sulcus, posterior boundary: postcentral sulcus, medial boundary: midline, lateral boundary: lateral sulcus), and descended through the corona radiata, the posterior limb of the internal capsule, the cerebral peduncle of the midbrain, the anterior pons, and the anterior medulla along the known pathway of the PT. By contrast, in thirty-four (18.3%, right hemisphere: 20, left hemisphere: 14) of the 186 hemispheres, APTs separated from the PT at the upper midbrain level, descended through the medial lemniscus from the midbrain to the pons, and then rejoined with the PT at the upper medulla. Nine (26.5%) of the 34 APTs were found to originate from the primary somatosensory cortex (anterior boundary: central sulcus, posterior boundary: postcentral sulcus, medial boundary: midline, lateral boundary: lateral sulcus) without a primary motor cortex (anterior boundary: precentral sulcus, posterior boundary: central sulcus, medial boundary: midline, lateral boundary: lateral sulcus) origin.

Mean values of fractional anisotropy (FA), mean diffusivity (MD), tract volume (the number of voxels), and incidence of the APT are summarized in table 1. FA value and tract volume of the APT were lower than those of the PT. There were significant differences in terms of FA (*t *= 11.86, *p *< 0.001) value and tract volume (*t *= 11.28, *p *< 0.001) between the PT and the APT in the independent t-test, (*P *< 0.05). Likewise, we found significant difference in FA (Pearson's correlations: *r *= -0.163, *P *= 0.015) value between the PT and the APT in one-way analysis of covariance (ANCOVA; age) (*P <*0.05). However, no difference in MD (Pearson's correlations: *r *= 0.146, *P *= 0.03) value was observed in both the independent t-test and ANCOVA (*P >*0.05). We did not observe any differences in the values of FA (PT: *t *= -0.84, *p *= 0.40 APT: *t *= -0.49, *p *= 0.62), MD (PT: *t *= 1.23, *p *= 0.22 APT: *t *= 1.63, *p *= 0.11, tract volume (PT: *t *= -0.83, *p *= 0.41 APT: *t *= -0.12, *p *= 0.90), and incidence of the APT between the right and left hemispheres in either the PT or the APT (*P *> 0.05).

**Table 1:** Results of diffusion tensor imaging parameters and incidence of the pyramidal tract and aberrant pyramidal tract.

		Right	Left	Total
Pyramidal tract	Fractional anisotropy	0.58 (± 0.02)	0.59 (± 0.02)	0.59 (± 0.02)
	Mean diffusivity	0.82 (± 0.03)	0.82 (± 0.04)	0.82 (± 0.04)
	Tract volume	580.72 (± 188.13)	605.1 (± 209.99)	592.91 (± 199.19)
	Incidence	93 (100%)	93 (100%)	186 (100%)
	
Aberrant Pyramidal tract	Fractional anisotropy	0.53 (± 0.02)	0.54 (± 0.03)	0.53 (± 0.02)
	Mean diffusivity	0.81 (± 0.03)	0.80 (± 0.02)	0.81 (± 0.03)
	Tract volume	189.25 (± 136.64)	195.07 (± 135.44)	191.65 (± 134.10)
	Incidence	20 (21.5%)	14 (15.0%)	34 (18.3%)

Values indicate mean (± standard deviation). Mean diffusivity × 10^-3^(mm^2^/s).

Incidence (percent).

## Discussion

In the current study, using DTT, we investigated the characteristics of the APT in comparison with the PT. We found the following three results. First, the APT existed in 18.3% of the hemispheres of the normal human brain. Second, although all of the PTs were found to originate from SM1, 26.5% of the APTs were found to originate from the primary somatosensory cortex without a primary motor cortex origin. Third, values of FA and tract volume for the APT were lower than those of the PT, with no difference in MD value. The FA value represents the degree of directionality of microstructures (e.g., axons, myelin, and microtubules), and the MD value indicates the magnitude of water diffusion [15-17]. In contrast, the tract volume was determined by the number of voxels contained within the neural tract [18]. Changes in DTI parameters observed in the APT, that is, decreased FA value and tract volume with unchanged MD value, suggest less directionality and fewer neural fibers than in the PT.

Like this study, several other studies have reported on the incidence and courses of the APT [3,6,11,12]. In 2001, using the modified Bielschowsky stain, Yamashita and Yamamoto [6] investigated the incidence and details of the course of the APT in 150 consecutive autopsied human brains. They found that all of the 150 brains examined, with the exception of one brain with holoprosencephaly, showed the APT, and reported that the course of the APT left the PT within the cerebral peduncle and then passed into the medial lemniscus of the pons through the upper medulla. In 2009, Hong et al [3] reported that the APT existed in 5 (17.9%) of the 28 hemispheres of normal subjects and that the APT descended through the medial lemniscus from the midbrain to the pons, and then entered into the PT at the upper medulla. Recently, several studies have suggested that the APT may contribute to motor recovery in stroke [1,2,11,12]. In 2009, Jang SH reported a patient whose motor function appeared to have recovered via an APT following a pontine infarct located in the PT area [11]. In 2010, Lindenberg et al. demonstrated that patients with alternate motor fibers in the brainstem showed better motor outcome among 35 patients with middle cerebral artery infarcts [12]. However, they did not clarify that the alternate motor fibers were APTs. In 2011, two patients were reported with midbrain infarct or corona radiata, respectively, that showed motor recovery via APT [13,14]. As for the incidence of APT existence, our results coincide with those of Hong's DTT study and the course of the APT was similar with that of all previous studies [3,6,11,12].

## Conclusions

We identified the APT and reported on the characteristics of the APT in comparison with the PT in the human brain. We found that the APT has the different characteristics of less directionality, fewer neural fibers, and less origin from the primary motor cortex than the PT. To the best of our knowledge, this is the first DTT study to report on the detailed characteristics of the APT in comparison with the PT in the human brain. We believe that the methodology and results of this study would be helpful in research on the APT in the human brain. However, limitation of DTI should be considered [19-21]. Even though DTI is a powerful anatomic imaging tool that can demonstrate the gross fiber architecture, limitation of DTI such as partial-volume effects which are non-trivial problems in interpreting of diffusion-weighted signal could influence the tract including the DTI parameters such as FA and MD [19-21]. In detail, the fiber bundles which are passing through narrow area could lead to dispersed patterns of low confidence connections downstream of the bottleneck. Therefore, the small neural tract such as the APT can be affected by partial volume effect [19]. Further studies of the clinical significance in relation to brain development and motor recovery following brain injury would be invited.

## Methods

### Subjects

We recruited ninety-three healthy right-handed subjects (males: 53, females: 40, mean age: 41.51 years, range: 20-78 years) with no previous history of neurological, physical, or psychiatric illness. The Edinburg Handedness Inventory was used for evaluation of handedness [22]. All subjects understood the purpose of the study and provided written, informed consent prior to participation. The study protocol was approved by our local Institutional Research Board.

### Diffusion Tensor Tractography

A 6-channel head coil on a 1.5 T Philips Gyroscan Intera (Philips, Ltd, Best, The Netherlands) with single-shot echo-planar imaging was used for acquisition of DTI data. For each of the 32 non-collinear diffusion sensitizing gradients, we acquired 67 contiguous slices parallel to the anterior commissure-posterior commissure line. Imaging parameters were as follows: acquisition matrix = 96 × 96; reconstructed to matrix = 128 × 128 matrix; field of view = 221 × 221 mm^2^; TR = 10,726 ms; TE = 76 ms; parallel imaging reduction factor (SENSE factor) = 2; EPI factor = 49; *b *= 1000 s/mm^2^; NEX = 1; and a slice thickness of 2.3 mm (acquired isotropic voxel size 2.3 × 2.3 × 2.3 mm^3^). Removal of eddy current-induced image distortions was performed at the Oxford Centre for Functional Magnetic Resonance Imaging of Brain Software Library (FSL; http://www.fmrib.ox.ac.uk/fsl) using affine multi-scale two-dimensional registration [23]. DTI-Studio software (CMRM, Johns Hopkins Medical Institute, Baltimore, MD, USA) was used for evaluation of the PT and APT [24]. Fiber tracking was based on the fiber assignment continuous tracking algorithm (FACT) and a multiple regions of interest (ROIs) approach. We selected two ROIs for the PT on the color map (blue: superioinferior orientation, red: mediolateral orientation, green: anteroposterior orientation). First ROI was placed on the posterior limb of the internal capsule. Second ROI was given on the PT area of the anterior medulla (portion of anterior blue color) with the option of AND operation with first ROI [3]. By contrast, for reconstruction of the APT, we selected three ROIs on the color map with the option of AND operation. First ROI was placed on the posterior limb of the internal capsule. Second ROI was given on the PT area of the anterior medulla (portion of anterior blue color) and additional ROI was placed on the isolated APT area (behind the posterior transpontine fiber (red) at the pontine level) [3]. Fiber tracking was initiated at the center of any voxel with a FA > 0.2 and ended at a voxel with a FA of < 0.2 and a tract turning-angle of < 60 degrees. Values of FA, MD, and tract volume for the PT and APT were measured.

### Statistical analysis

SPSS software (v.15.0; SPSS, Chicago, IL) was used for data analysis. Independent t-test was used for determination of the differences in values of DTI parameters (FA, MD, and tract volume) between the right and left hemispheres, and between the PT and the APT. In addition, we performed pearson's correlations in FA, MD, and tract volume with age before performing the ANCOVA because it was necessary to confirm correlations to perform ANCOVA. As a result, we found that there were significant correlations in FA (Pearson's correlations: *r *= -0.163, *P *= 0.015) and MD (Pearson's correlations: *r *= 0.146, *P *= 0.03) with age. We performed ANCOVA to determinate the differences in values of FA and MD with age. However, we could not perform ANCOVA for tract volume because there was no significant correlation in tract volume with age (Pearson's correlations: *r *= -0.037, *P *= 0.583). For comparison with incidence of the APT, the chi-square test was performed between the right and left hemispheres. The significant level of the *P *value was set at 0.05.

## Lists of Abbreviations

APT: aberrant pyramidal tract; DTI: diffusion tensor imaging; DTT: diffusion tensor tractography; FA: fractional anisotropy; FACT: fiber assignment continuous tracking algorithm; MD: mean diffusivity; PT: pyramidal tract; ROI: regions of interest; SM1: primary sensori-motor cortex; SNR: signal-to-noise ratio.

## Authors' contributions

HGK, data acquisition, manuscript development and oversight, technical support, data acquisition, and manuscript writing. SMS, research design, data acquisition, MCC, technical support, data acquisition, SK, technical support, data acquisition, YHK, technical support, data acquisition, SHJ, conceiving and designing the study, manuscript development, funding, and manuscript authorization. all authors read and approved the final manuscript

**Figure 1:**
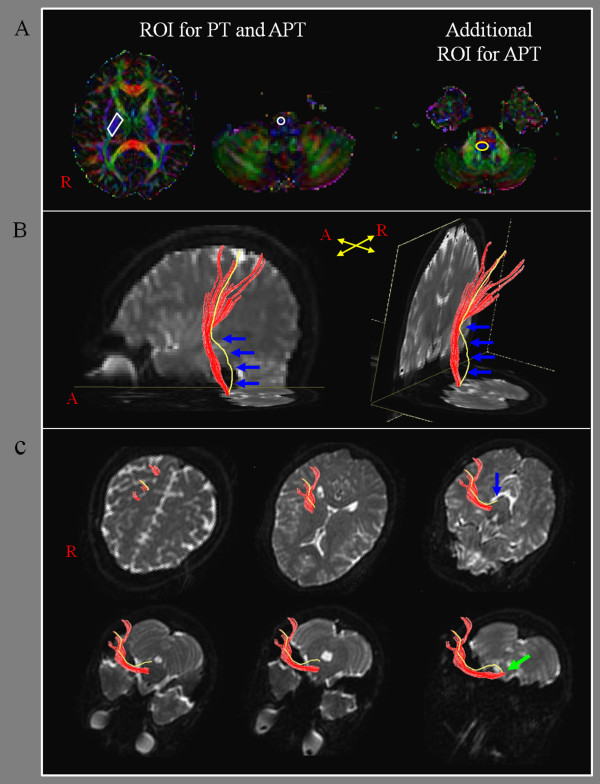
**Results of diffusion tensor tractography for the pyramidal tract (PT) and the aberrant pyramidal tract (APT)**. (A) For the reconstruction of the PT, two regions of interest (ROIs) were placed on the posterior limb of the internal capsule and the PT area of the anterior medulla (portion of anterior blue color) on the color map. By contrast, for the reconstruction of the APT, three ROI were given on the posterior limb of the internal capsule, the PT area of the anterior medulla (portion of anterior blue color), and additional ROI was placed on the isolated APT area (behind the posterior transpontine fiber at the pontine level). (B) the PT and APT were constructed in the right hemisphere (red: the PT, yellow: the APT). The APT descended along the known pathway of the PT to the posterior limb of the internal capsule and then descended through the medial lemniscus from the midbrain to the pons (blue arrows). (C) The pathways of the PT and the APT are shown at the axial views (blue arrow: the APT was separated from the PT at the upper midbrain level, green arrow: the APT rejoined with the PT at the upper medulla level).
